# Clinical features, management, and outcomes of posterior capsule rupture during phacoemulsification surgery

**DOI:** 10.5935/0004-2749.2024-0328

**Published:** 2025-06-24

**Authors:** Efe Koser, Burcu Kemer Atik, Merve Emul, Sibel Ahmet, Nilay Kandemir Besek, Mehmet Ozgur Cubuk, Ahmet Kirgiz

**Affiliations:** 1 University of Health Sciences, Beyoglu Eye Training and Research Hospital, Istanbul, Turkey

**Keywords:** Cataract extraction, Phacoemulsification, Posterior capsule rupture, Corneal edema, Risk factors, Postoperative complications, Intraoperative complications

## Abstract

**Purpose:**

Posterior capsule rupture is defined as an intraoperative posterior capsule tear resulting in vitreous loss. This study aimed to analyze the clinical characteristics, preoperative risk factors, intraoperative management strategies, and postoperative complications associated with posterior capsule rupture during phacoemulsification surgery.

**Methods:**

This was a retrospective observational cohort study of the medical records for 25,224 phacoemulsification surgeries performed at our tertiary eye care center between 2017 and 2022. We collected and collated the demographic characteristics and clinical findings of the patients in our cohort. Intraoperative management strategies and postoperative outcomes over a 1-year followup period were also recorded.

**Results:**

Posterior capsule rupture occurred in 351 eyes (351 patients), giving an overall posterior capsule rupture rate of 1.3%. The mean patient age was 68.6 ± 10.8 years. Pseudoexfoliation syndrome, mature cataracts, brown cataracts, and surgery performed by a resident were identified as risk factors for posterior capsule rupture (p<0.05 for each; the risk ratios were 2.70, 2.15, 2.44, 1.34, respectively). The most common intraoperative complications were dislocated lens fragments in the vitreous (8%) and iris damage (7.1%). The mean best-corrected visual acuity improved from 1.31 ± 0.84 (logMAR) postoperatively to 0.51 ± 0.56 at the end of the 1-year follow-up period (p<0.001). Corneal edema (55.6%) and elevated intraocular pressure (33.3%) were the most common early postoperative complications. Persistently elevated intraocular pressure (11.1%) and cystoid macular edema (5.1%) were the most common late postoperative complications.

**Conclusion:**

Posterior capsule rupture is a common complication of phacoemulsification surgery that requires prolonged postoperative follow-up and a multidisciplinary approach. Despite the increased incidence of complications when rupture occurs, appropriate intraoperative and postoperative management can lead to satisfactory visual outcomes.

## INTRODUCTION

Cataracts are the leading cause of reversible blindness worldwide^(1)^, and phacoemulsification surgery is the most common procedure for its treatment^(2)^. Despite advancements in surgical techniques, posterior capsule rupture (PCR) remains a significant and potentially sight-threatening complication^(3^-^6)^. When it occurs, PCR increases the complexity of the procedure, particularly when vitreous prolapse occurs. It also significantly increases the risk of severe postoperative complications such as retinal detachment, macular edema, uveitis, glaucoma, and intraocular lens (IOL) dislocation^(4^-^11)^. These complications can compromise both the shortand long-term visual outcomes of the patient.

Previous studies have identified various preoperative risk factors for PCR. These include pseudoexfoliation syndrome (PEX), diabetic retinopathy, and high myopia. These studies have also considered the intraoperative challenges presented by PCR, particularly for less-experienced surgeons^(4^,^7^,^8^,^10)^. However, despite the existing literature, gaps remain in our understanding of the interplay between the demographic, clinical, and surgical factors that contribute to PCR and its outcomes.

An additional area that requires further exploration is the implications of PCR for postoperative management and recovery. Proper handling of PCR-related complications, including timely surgical intervention and appropriate follow-up care, can significantly influence the prognosis for the patient’s vision. This study will examine these aspects of PCR. We aim to address both the factors that contribute to its occurrence and the most effective strategies with which to mitigate its long-term impact.

To achieve this, we analyzed a large cohort of patients who developed PCR during phacoemulsification surgery at a tertiary eye care center. Specifically, we evaluate the demographic characteristics, clinical features, preo-perative risk factors, intraoperative and postoperative management strategies, and one-year outcomes associated with PCR. By identifying critical risk factors and complications, we hope to contribute to the optimization of preoperative planning, surgical safety, and overall patient outcomes.

## METHODS

This study was approved by Turkey’s National Ethics Council with 21/3 approval number and was conducted in accordance with the tenets of the 1964 Declaration of Helsinki and its later revisions. In this retrospective single-center study, we searched the medical records of 25,224 patients who underwent cataract surgery between January 2017 and December 2022. Patients who suffered intraoperative PCR, who were aged over 40 years, and who were seen for a regular one-year follow-up after the procedure were included in the study. Patients with traumatic cataracts, preoperative lens dislocation, and those in whom the surgeon chose to perform posterior capsulorhexis were excluded.

Detailed information was obtained from the patients regarding demographic characteristics (age, sex, etc.), systemic and ocular comorbidities and medications, previous ocular surgeries, and ocular characteristics. Each patient received a comprehensive ophthalmic examination at all visits to our center. This included measurements of uncorrected visual acuity (UCVA), best-corrected visual acuity (BCVA), intraocular pressure (IOP) (measured with applanation tonometry), anterior segment examination, and dilated fundus examination. The visual acuities were measured using the Snellen chart and subsequently converted to logarithm of the minimum angle of resolution (logMAR) units. Preoperative cataract grading was performed using the Emery-Little classification system^(12)^. Regression analysis and chi-square test were performed on potential preoperative risk factors, and the relative risk was calculated for each.

In all instances, the surgeon performing the procedure was determined during the preoperative evaluation based on surgical experience and the complexity of the case. All procedures performed by residents were supervised by a consultant. When PCR occurred, the management of the complication depended on surgical experience and the stage at which the rupture occurred. If a resident had performed fewer than 100 surgeries or the complication required expert intervention, it was managed directly by the supervising consultant. Intraoperative posterior capsule tear with vitreous loss was defined as PCR. If PCR occurred, either the phacoe-mulsification surgery was continued, or extracapsular cataract extraction (ECCE) was performed, depending on the stage of surgery and the amount of remaining lens material. All of the patients underwent anterior vitrectomy. Where appropriate, the PCR flap was converted to a posterior capsulorhexis, and a single-piece IOL was implanted in the capsular bag. In patients without capsular support, a three-piece IOL was placed in the ciliary sulcus when sulcus support was available. Otherwise, the IOL was implanted using scleral fixation. Depending on the surgical time, complications, and patient compliance, IOL implantation was performed either during the same surgery or as a separate subsequent procedure. The Sanders Retzlaff Kraff theoretical (SRK/T) formula was used for IOL calculation with normal and long axial lengths^(13)^, the Hoffer Q formula with short axial lengths^(14)^, and the Shammas PL formula in cases with a history of refractive surgery^(15)^.

All patients were examined on postoperative day 1, week 1, month 1, month 3, month 6, and year 1. The frequency of postoperative visits was increased for patients with additional complications, depending on their condition. Postoperatively, all patients were prescribed moxifloxacin drops, to be used four times a day for 4 weeks, and prednisolone acetate drops, to be used six times a day for a maximum of 2 months, and nepafenac eye drops, to be used four times a day for 4 weeks. The frequency of steroid drop use was adjusted based on factors such as the presence of corneal edema or IOP. The surgeon’s experience level, the stage of surgery at which PCR occurred, the IOL implantation method, and any intraoperative and/or postoperative complications were recorded. The first month following surgery was classed as the early postoperative period, and beyond the first month as the late postoperative period. IOP greater than 21 mmHg was deemed to be elevated IOP and recorded as such.

### Statistical analysis

SPSS Statistics for Windows, v.22 (IBM Corp., Armonk, NY, USA) software was used to perform all statistical analyses. Continuous data were reported as mean, standard deviation (SD), and range. Categorical data were reported as frequency and percentage. The normality of data distribution was assessed using the Kolmogorov-Smirnov test. Normally distributed preoperative and postoperative BCVA values were analyzed using paired sample t-tests. Repeated measures ANOVA or Friedman tests were utilized to compare normally and nonnormally distributed repeated measurements, respectively. One-way ANOVA and Kruskal-Wallis tests were used to compare respective normally and nonnormally distributed data from multiple groups. In all analyses, the significance level was 95%, and the results were deemed statistically significant if p-values were <0.05.

## RESULTS

### Demographic characteristics of the PCR patients

PCR occurred in 351 (199 right, 152 left) eyes of 351 (202 male, 149 female) patients. The mean age was 68.6 ± 10.8 years. The mean follow-up was 13.86 ± 7.68 months. The most frequent concomitant systemic disease was diabetes mellitus (DM), and the most frequent ocular comorbidity was PEX. The preoperative characteristics, previous ocular surgeries, and cataract types of the patients are summarized in table 1. The most common cataract type was stage 1-2 nuclear cataracts in 106 (30.2%) eyes, followed by posterior subcapsular cataracts in 90 (25.6%) eyes.

**Table 1:** Demographic and baseline clinical characteristics of patients who suffered posterior capsule rupture during cataract surgery

Characteristic	n (%)
Age (years) mean ± SD	68.6 ± 10.8
Sex	
Male	202 (57)
Female	149 (43)
Laterality	
Right	199 (56)
Left	152 (44)
Surgeon experience level	
Resident	144 (41)
Consultant	207 (59)
Comorbidities	
DM	122 (34.8)
PEX	90 (25.6)
History of tamsulosin usage	34 (9.7)
High myopia	12 (3.4)
Previous ocular surgeries	
Pars plana vitrectomy	8 (2.2)
Trabeculectomy	4 (1.1)
Corneal refractive surgery	2 (0.6)
Cataract type	
Stage 1-2 nuclear sclerotic	106 (30.2)
Stage 3-4 nuclear sclerotic	62 (17.6)
Posterior subcapsular	90 (25.6)
Cortical	26 (4.6)
Mature	45 (12.8)
Brown	16 (4.6)
Posterior polar	5 (1.4)
Intumescent	1 (0.3)

Categorical data are given in number (%).

DM= diabetes mellitus; PEX= pseudoexfoliation syndrome; SD= standard deviation.

### PCR incidence, risk factors, and management

The overall PCR rate among the 25,224 patients who underwent cataract surgery at our center during the period studied was 1.3%. PEX, mature cataracts, and brown cataracts were identified as preoperative risk factors (p<0.001 for each). PCR occurred in 90 (3.2%) of the 2,770 eyes with PEX (risk ratio = 2.70). PCR was detected in 45 (2.8%) of the 1,570 eyes with mature cataracts (risk ratio = 2.15). Among the 468 eyes with brown cataracts, PCR occurred in 16 eyes (3.4%) (risk ratio = 2.44).

PCR occurred in 144 (1.6%) of the 8,675 surgeries performed by residents and in 207 (1.2%) of the 16,648 surgeries performed by consultants. Thus, the PCR rate was significantly lower in those performed by consultants (p=0.006, risk ratio = 0.74). However, there was no significant difference in the stage at which PCR occurred, vision outcomes, or incidence of complications between surgeries performed by residents and consultants (p>0.05 for each). Table 2 presents a comparison between residents and consultants based on the stage at which PCR occurred and the number of complications arising at each stage. PCR was seen most often during the phacoemulsification phase (221 eyes, 63%), followed by the irrigation and aspiration (I&A) phase (82 eyes, 23.4%). Although the final (1-year) BCVA was numerically worse in patients who developed PCR in the earlier stages of phacoemulsification (capsulorhexis and sculpting), there was no statistically significant difference between the final BCVA of patients who developed PCR at different stages (p=0.67). There was also no significant relationship between the stage at which PCR occurred and the incidence of either early or late postoperative complications (p=0.70, p=0.63, respectively). Phacoemulsification surgery was converted intraoperatively to ECCE in 14 (4%) of the eyes in which PCR occurred. IOL implantation could not be performed during the same surgery in 94 (26.8%) eyes. Among the 257 (73.2%) eyes that underwent IOL implantation during the same surgery, the implantation location was the capsular bag in 36 (10.3%) and the ciliary sulcus in 197 (56.1%). Scleral fixation was used in the remaining 24 (6.8%) eyes. Of the patients who remained aphakic after the first surgery, 71 (20.2%) underwent scleral fixation-54 (15.3%) with the Z suture method and 17 (4.8%) with the Yamane method-while 15 (4.2%) received sulcus IOL placement. These subsequent procedures took place, on average, 2.77 ± 2.10 (1-10) months later.

**Table 2:** Comparative outcomes of cataract patients according to the surgical stage at which PCR occurs

Stage of surgery	Resident	Consultant	Total	BCVA (logMAR)	Dislocated lens fragments n (%)	Early postoperative complications n (%)	Late postoperative complications n (%)
Capsulorhexis	5 (3.4%)	8 (3.8%)	13 (3.7%)	0.67 ± 0.88	1 (3.5)	8 (3.6)	3 (3.5)
Sculpting	3 (2%)	3 (1.4%)	6 (1.7%)	0.95 ± 0.76	2 (7.1)	4 (1.8)	1 (1.1)
Phacoemulsification	77 (53.5%)	144 (69.6%)	221 (63%)	0.39 ± 0.46	19 (67.8)	143 (65.2)	53 (63)
Irrigation and aspiration	45 (31.2%)	37 (17.9%)	82 (23.4%)	0.43 ± 0.58	6 (21.4)	49 (22.3)	23 (27.3)
IOL implantation and OVD removal	14 (9.7%)	15 (7.4%)	29 (8.2%)	0.38 ± 0.60	-	15 (6.8)	4 (4.7)
**Total**	**144**	**207**	**351**	**0.42 ± 0.53**	**28**	**219**	**84**

The columns are presented such that each totals 100%.

BCVA= best-corrected visual acuity; IOL= intraocular lens; OVD= ophthalmic viscosurgical devices; PCR= posterior capsule rupture.

### Intraoperative and postoperative complications

The most common intraoperative complication was dislocated lens fragments in the vitreous, which occurred in 28 (8%) eyes. Of these, nine (7.9%) eyes required *pars plana* vitrectomy (PPV). PPV was performed in the same surgery in two (0.05%) eyes due to the large size of the dislocated fragment. In the remaining seven (1.9%), PPV was performed an average of 1.20 ± 0.96 months later due to elevated IOP and intraocular inflammation. The most common early postoperative complication was corneal edema, which was seen in 195 (55.6%) eyes. This was followed by IOP elevation in 117 (33.3%) eyes and vitreous hemorrhage (VH) in seven (1.9%) eyes. While VH resolved spontaneously in six (1.7%) eyes with conservative management during follow-up, PPV was required in one (14.3%). On postoperative day 1, two (0.5%) eyes had hyphema, and one (0.2%) had a fibrin membrane in the anterior chamber, all of which were managed with topical medication.

In the late postoperative period, persistent IOP elevation was observed in 39 (11.1%) eyes. Two (0.5%) eyes required glaucoma surgery. Transscleral diode laser cyclophotocoagulation was performed 3 months postoperatively in one of these, while trabeculectomy was performed 6 months postoperatively in the other. Central corneal edema persisted for more than 1 month in four (1.1%) eyes. Topical treatment was continued in these four. In two, the corneal edema regressed with treatment, whereas corneal endothelial dysfunction became permanent in the other two. One of these regressed slightly with topical treatment, and visual acuity reached a satisfactory level; the other underwent Descemet’s membrane endothelial keratoplasty.

The most frequent vision-threatening late postoperative complication was cystoid macular edema (CME) in 18 (5.1%) eyes. In 13, this was resolved with topical anti-inflammatory therapy. In those requiring further treatment, three eyes received subtenon triamcinolone acetate injections in month 1, and two received intravitreal dexamethasone implantation in month 3. During follow-up, retinal detachment (RD) occurred in nine (2.5%) eyes and tractional RD in one (0.2%) eye. RD occurred, on average, 9.87 ± 4.31 (2-15) months after the initial surgery. All of the intraoperative and postoperative complications are summarized in table 3.

**Table 3:** Intraoperative and postoperative complications of patients who suffered posterior capsule rupture during cataract surgery

Complication type	n (%)
Intraoperative complications	
Dislocated lens fragments into the vitreous	28 (8)
Iris damage IOP elevation and corneal edema	25 (7.1) 2 (0.5)
Postoperative complications	
Early^†^	
Corneal edema	159 (55.6)
IOP elevation	117 (33.3)
Vitreous hemorrhage	7 (1.9)
Hyphema	2 (0.5)
Fibrin membrane	1 (0.2)
Endophthalmitis	1 (0.2)
Late	
Persistent IOP elevation	39 (11.1)
Persistent corneal edema	4 (1.1)
CME	18 (5.1)
RD	9 (2.5)
IOL dislocation	5 (1.4)
ERM	3 (0.8)
RVO	1 (0.2)
TRD	1 (0.2)

† indicates the first postoperative month.

CME= cystoid macular edema; ERM= epiretinal membrane; IOL= intraocular lens; IOP= intraocular pressure; RD= retinal detachment; RVO= retinal vein occlusion; TRD= tractional retinal detachment.

BCVA= best-corrected visual acuity.

### Vision outcomes

**Figure 1:**
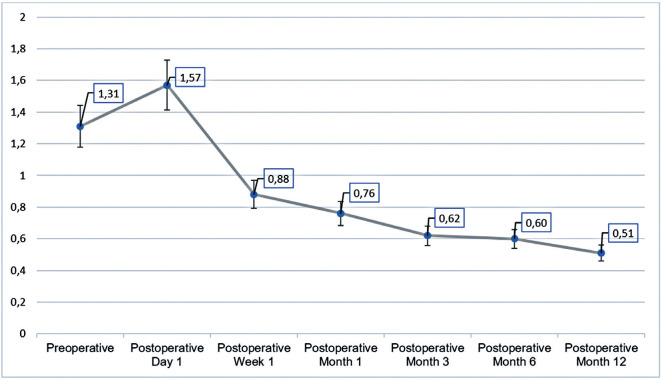
Graph showing the BCVA values of patients who suffered posterior capsule rupture during cataract surgery.

The course of BCVA among the cohort during the follow-up period is shown in figure 1. The mean preoperative BCVA (logMAR) was 1.31 ± 0.84. This had worsened to 1.57 ± 0.94 on postoperative day 1 and then improved to 0.88 ± 0.62 at week 1, 0.76 ± 0.65 at month 1, 0.62 ± 0.59 at month 3, 0.60 ± 0.60 at month 6, and 0.51 ± 0.56 at the final (1 year) follow-up (p<0.001). While no significant difference in BCVA was observed between preoperative values and postoperative day 1 values (p=0.14), a statistically significant improvement in BCVA was observed at later postoperative visits compared with the preoperative BCVA (p<0.001 at each time point). The proportion of patients who achieved a final BCVA of 0.30 or better was 79.4% (279 of 351).

## DISCUSSION

The history of cataract surgery has been a journey of evolving techniques, from intracapsular cataract extraction to the more advanced phacoemulsification method. The minimally invasive nature of phacoemulsification is the primary reason for its widespread adoption. However, learning this technique is challenging. PCR is perhaps the most feared event during this learning curve. As highlighted in previous research, it is the most common complication of phacoemulsification^(3^-^6)^. Studies of PCR have generally had short followup periods (up to 6 months) due to large numbers of patients lost to follow-up^(16^-^18)^. The current population-based study with a one-year follow-up and a large sample was designed to provide valuable information on the incidence, risk factors, and management of PCR.

Several risk factors for PCR have previously been identified. The most commonly reported are advanced patient age (especially >80 years), diabetic retinopathy, PEX, and high myopia^(4^,^7^,^8^,^10^,^19^-^25)^. In our study, the mean age of patients with PCR was 68.6 years, 34.8% of the patients were diabetic, and 25.6% had PEX. These results confirm that PCR appears to be more frequent in these patient groups, and more care should be taken with these patient subsets.

Among the 25,224 phacoemulsification surgeries performed at our institution during the study period, the PCR rate was 1.3%. This is consistent with the previously reported rates of 0.6-5.2%^(3^-^5^,^8^,^11^,^26)^.

Segers et al.^(4)^, studied a large population of 2,853,376 patients and encountered PCR in 31,749 (1.1%) of cataract surgeries. This rate ranged from 0.60% to 1.65%, with a decreasing trend over 10 years. Another study of 1,200 cases by Bai et al.^(5)^ reported a PCR rate of 4.5% in the first 100 surgeries performed by six residents, which declined to 2% in the second 100 cases. Hence, the PCR rate decreases as surgical experience increases, as might be expected^(5^,^7^,^8)^. However, Barreto et al.^(26)^, found no difference in the rate of PCR between residents and experienced surgeons. They attribute this to adequate supervision during procedures. Nonetheless, the procedures performed by residents in the present study had a significantly higher incidence of PCR than those performed by consultants, despite supervision in all instances. In addition, since the consultants in our institution handled more difficult and complicated cases, the risk of PCR in resident cases may be higher than we have reported in facilities where this is not the protocol. Given the challenging nature of phacoemulsification surgery, it is not surprising that less-experienced surgeons will encounter PCR more frequently. Still, the necessary precautions should be taken and awareness raised to keep the PCR rates to a minimum.

The surgical stage at which PCR most frequently occurs varies between studies. In studies of 127 PCRs by Basti et al.^(27)^ and 77 PCRs by Thanigasalam et al.^(28)^, PCR occurred most frequently during the I&A stage (with rates of 52.4% and 35.2%, respectively). However, studies with larger cohorts have found PCR to occur most often during the phacoemulsification stage, with a rate of 42.2% in a study by Ang and Whyte (2,727 cases, 45 PCRs, 1.7% PCR rate)^(29)^ and 59.6% in the study of Seng-Ei TI et al. (48,778 cases, 887 PCRs, 1.8% PCR rate)^(16)^. In the present study, PCR developed most commonly in the phacoemulsification stage (63%). Although Seng-Ei TI et al. did not directly examine the relationship between the stage of PCR occurrence and BCVA, over 90% of their patients achieved BCVA of 0.30 and better after PCR, regardless of stage^(16)^. In our study, we found no statistical relationship between the surgical stage of PCR and the final BCVA. However, there was a nonsignificant trend toward worse final BCVA values in patients whose PCR occurred during earlier surgical stages. This may be due to the difficulty in extracting the nucleus when PCRs develop while the nuclear material is in the eye. We also found that the stage at which PCR occurred had no statistically significant relationship with the incidence of early and late postoperative complications. These results indicate that, regardless of the stage at which PCR occurs, good results can be achieved with proper management.

Dislocation of lens fragments into the vitreous cavity is the most frequent intraoperative complication subsequent to PCR. Ang and Whyte^(29)^ reported a 22.2% (10 of 45 PCRs) rate of PCR-associated dislocated lens fragments into the vitreous, three (6.7%) of which underwent PPV. However, Ti et al.^(16)^ reported a 3.9% (35 of 887 PCRs) rate of dislocated lens fragments, with six (0.6%) requiring PPV. In our study, the dislocated lens fragment rate was 8%. Although this is compatible with other rates reported in the literature, it is relatively high. We believe this is because it is the policy at our institution to clearly record even the smallest lens fragment dislocation for postoperative observation. PPV was required in 32% of our lens fragment patients. It is remarkable that PPV is not required by most of these patients and can be managed with a conservative approach. When the retained lens material is soft and does not cause inflammation, patients can be followed closely without significant intervention^(16^,^29)^. However, careful intraoperative monitoring of any fragments that fall into the vitreous is essential, with particular attention to the size and hardness of the fragments. As highlighted by Vanner et al.^(30)^ same-day PPV in cases with large dislocated fragments may be crucial to satisfactory long-term outcomes. If the surgical center is unable to perform PPV, this should be recognized during follow-up, and no time should be lost. Seng-Ei TI et al.^(16)^ have reported a 1.8% rate, and Ang and Whyte^(26)^, a 2.2% rate of PCR-related CME. The authors of these studies suggest that their rates may have been low due to their short follow-up periods. Kumar et al.^(31)^ have reported a PCR-related CME rate of 10.71%. A review by Blomquist and Rugwani documents CME incidences of up to 21% following vitreous loss in ECCE cataract surgery, with a mean follow-up of 11 months^(17)^. In our study, the CME rate was 5.1%, consistent with the literature. CME should be considered when there is no pathology of the cornea or lens to explain visual impairments in the patient. As patients with PCR are known to have an increa-sed risk of CME, they should be followed up for longer.

Although RD is a rare postoperative complication in standard phacoemulsification surgery, the risk is increased in patients with PCR^(32)^. In a large multicenter study by Day et al.^(11)^, patients with PCR demonstrated a 42-fold increased risk of RD in the 3 months following the procedure in which PCR occurred. They also found an eightfold increase in this risk of endophthalmitis. This latter is supported by a systematic review and meta-analysis that identified an increased risk of endophthalmitis associated with PCR^(18)^. In our study, the RD rate was 2.5%, and the endophthalmitis rate was 0.2%. The incidence rates of these significant complications increase in patients with PCR. Therefore, postoperative follow-up should be planned accordingly and patients should be informed in detail about these complications.

It is known that eyes complicated with PCR during cataract surgery have a significant risk of reduced visual acuity^(16^,^17^,^32)^. Yet, Ang and Whyte^(29)^ found that 84.4% of patients with PCR (45 PCRs in 2,727 cases) achieved a final BCVA of 0.30 or better. Similarly, Blomquist and Rugwani^(17)^ found that 77% (63 PCRs in 1,400 cases) achieved this BCVA. In our study, the rate was 79.4% (279/351). We also found significant improvements in BCVA over time, despite PCR, with the mean BCVA improving from 1.31 ± 0.84 preoperatively to 0.51 ± 0.56 at the end of the follow-up period. These results demonstrate that, when PCR occurs, good visual acuity can still be achieved with adequate management.

The main limitations of this study were its retrospective nature and the lack of a control group. The primary reason for our inability to create a control group was incomplete long-term data on postoperative outcomes and complications in our cohort since uncomplicated cases are routinely discharged from our hospital in the first postoperative month as it is a reference center with a high volume of patients. Another important limitation was that, due to the retrospective nature of the study, surgeon experience could not be classified in greater detail according to years of specialization or number of cases.

In conclusion, PCR is an unavoidable complication of phacoemulsification surgery. Our findings indicate that the incidence of PCR decreases with increased surgical experience. Those patients in whom PCR occurs should be followed up in a multidisciplinary manner for possible complications, and the potential need for additional surgeries should be kept in mind. With proper management, satisfactory visual and anatomical outcomes can be achieved.
